# Supplementary material for: Optimal measurement method for anterior instability on stress radiographs in anterior cruciate ligament tear: Considering the effect of static anterior tibial subluxation
Source: PLoS One. 2024 Sep 17;19(9):e0310428. doi: 10.1371/journal.pone.0310428 (PMC11407638; doi:10.1371/journal.pone.0310428)
Supplement: Attachment — Submitted filename: review.docx [file pone.0310428.s002.docx]

1.It is possible that ACL injuries may be combined with other conditions, such as knee osteoarthritis and synovitis. It is therefore important to consider whether the patients enrolled in this study were excluded from the disturbances caused by these possible co-morbidities. Were the patients enrolled in this study with ACL confounders excluded?

2.Whether the two measurements mentioned in the paper, "the traditional load-unload method" and "the load method", are routine in clinical practice.

3.This is a retrospective study, the control group of 28 healthy patients was matched to 340 patients who underwent surgical procedures other than ACL reconstruction, did the surgical operations the patients underwent interfere with the results of this study? These patients also underwent the same assessment components as ACL patients, are these measurements also routine in the clinic?

4.This study concludes,“In conclusion, the measurement method for anterior instability using load SSD showed superior diagnostic accuracy compared to the load-unload SSD method, which is susceptible to false negatives owing to static anterior tibial subluxation in ACL tear cases”,does static anterior tibial subluxation also influence the diagnosis of anterior instability in patients with ACL injuries when using the load-unload SSD method?
