# Supplementary material for: Optimal measurement method for anterior instability on stress radiographs in anterior cruciate ligament tear: Considering the effect of static anterior tibial subluxation
Source: PLoS One. 2024 Sep 17;19(9):e0310428. doi: 10.1371/journal.pone.0310428 (PMC11407638; doi:10.1371/journal.pone.0310428)
Supplement: Attachment — Submitted filename: loading_laxity_reviewer_answer_0814.docx [file pone.0310428.s004.docx]

We are grateful for the effort that the reviewer has put into the review process. Thanks to the detailed comments provided by the reviewer, we were able to correct the remaining typographical errors in this paper and further improve its overall quality. Our responses to the reviewer are provided below. Thank you again for your valuable feedback.

GENERAL

Reviewer #1: (No Response)

Reviewer #3: This is the second version of this manuscript that was resubmitted after considering the comments of previous 2 Reviewers. The article is about comparing 2 radiological methods for diagnosing the anterior instability in patients with ACL injuries using the Telos device: The load side to side difference (SSD) and the load unload (SSD). The authors found that the load SSD method has a higher diagnostic value specially in chronic cases. They aue this for the static anterior translation occurs in patients with chronic ACL Injuries.

The authors considered all comments of the previous reviewers and modified the article accordingly in a positive way; Yet there are still improvement potential in the article as follows :

Lines 26-28 (Abstract): the authors included 30 patients in acute group, 28 Patients in the chronic group and 30 patients in the control group. The sum should be 88 not 86 as the authors mentioned.

-> We have corrected this number to 88.

(Revised page #2/lines 26)

Line 90: the authors mentioned that they reviewed 486 patients operated in the their center to choose the included patients, but in the flow chart (Figure 1) they mentioned that the reviewed patients were 456 patients. Please unify this number.

-> The correct number is 456 patients. We have revised the text accordingly.

(Revised page #4/lines 91)

Line 252: the authors mentioned the abbreviation ALL. Please write it formal because it was not mentioned previously in the . I suppose they mean the anterolateral ligament.

-> We intended to refer to the anterolateral ligament. We have revised the text to include both the full term and the abbreviation.

(Revised page #10/lines 254)

Reviewer #4: Thank you very much for allowing me to review your manuscript. I appreciate the effort you have made performing this study and submitting it to plos one. I think this is appropriate for publishing.
